# Targeting the PI3K/AKT/mTOR pathway in lung cancer: mechanisms and therapeutic targeting

**DOI:** 10.3389/fphar.2025.1516583

**Published:** 2025-02-18

**Authors:** Min Qiang, Zhe Chen, Hongyang Liu, Junxue Dong, Kejian Gong, Xinjun Zhang, Peng Huo, Jingjun Zhu, Yifeng Shao, Jinazun Ma, Bowei Zhang, Wei Liu, Mingbo Tang

**Affiliations:** ^1^ Department of Thoracic Surgery, The First Hospital of Jilin University, Changchun, China; ^2^ College of Clinical Medicine, Jilin University, Changchun, China; ^3^ Department of Molecular Biology, Max Planck Institute for Infection Biology, Berlin, Germany; ^4^ Laboratory of Infection Oncology, Institute of Clinical Molecular Biology, Christian-Albrechts-Universität zu Kiel and University Hospital Schleswig-Holstein, Kiel, Germany; ^5^ Department of Thoracic and Cardiovascular Surgery, Severance Hospital, Yonsei University College of Medicine, Seoul, Republic of Korea; ^6^ Department of General Surgery, Capital Institute of Pediatrics’ Children’s Hospital, Beijing, China

**Keywords:** PI3K/Akt/mTOR pathway, lung cancer, PI3K inhibitors, Akt inhibitors, mTOR inhibitors, natural products, combined therapy

## Abstract

Owing to its high mortality rate, lung cancer (LC) remains the most common cancer worldwide, with the highest malignancy diagnosis rate. The phosphatidylinositol-3-kinase (PI3K)/protein kinase B (AKT)/mammalian target of rapamycin (mTOR) signaling (PAM) pathway is a critical intracellular pathway involved in various cellular functions and regulates numerous cellular processes, including growth, survival, proliferation, metabolism, apoptosis, invasion, and angiogenesis. This review aims to highlight preclinical and clinical studies focusing on the PAM signaling pathway in LC and underscore the potential of natural products targeting it. Additionally, this review synthesizes the existing literature and discusses combination therapy and future directions for LC treatment while acknowledging the ongoing challenges in the field. Continuous development of novel therapeutic agents, technologies, and precision medicine offers an increasingly optimistic outlook for the treatment of LC.

## 1 Introduction

**FIGURE 1:**
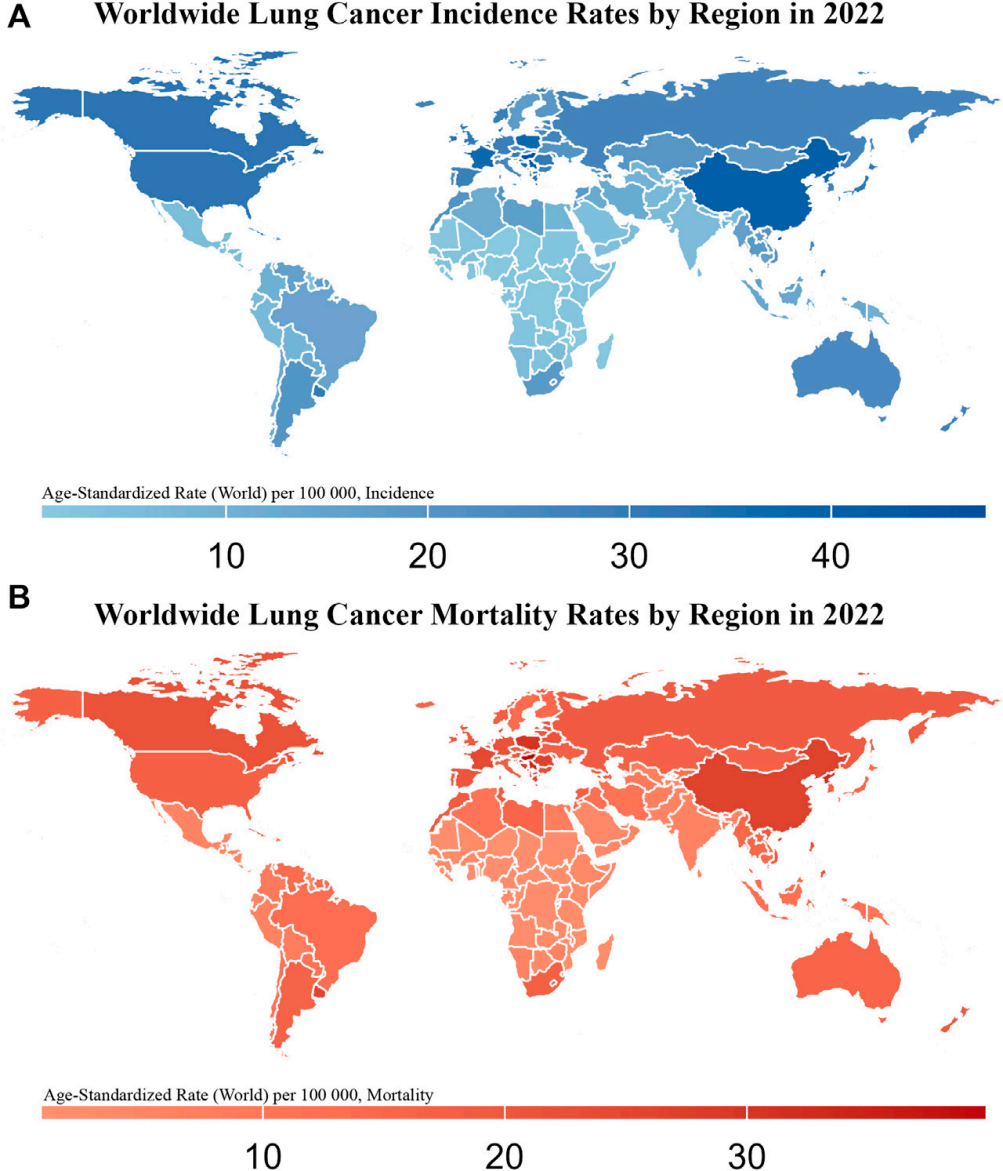
Worldwide Lung Cancer Incidence and Mortality Rates by Region in 2022 This figure consists of two maps showing the age-standardized incidence **(A)** and mortality rates **(B)** of lung cancer (per 100,000 population) across different regions of the world in 2022. The color gradient in **(A)**, from light blue to dark blue, represents increasing incidence rates, with darker blue regions indicating higher lung cancer incidence. In **(B)**, the color gradient from light red to dark red represents increasing mortality rates, with darker red regions indicating higher lung cancer mortality.

The International Agency for Research on Cancer (IARC) predicts that in 2022, lung cancer (LC) will have the highest global rates of incidence at 12.4% and mortality at 18.7% (Bray et al., 2024). The 2022 report from the China National Cancer Center indicated that the number of LC cases in China will increase to 1,060,600, with 733,300 deaths. LC remains the leading cause of both incidence and mortality (Figure 1) (Han et al., 2024). Smoking is the most significant risk factor that markedly increases the incidence of LC. In China, smoking is responsible for approximately 44.7% of reported LC deaths in males and 6.4% in females (Xia et al., 2019). Based on the morphological characteristics of LC cells, LC is classified into small-cell lung cancer (SCLC, approximately 15%) and non-small-cell lung cancer (NSCLC, approximately 85%) (Li et al., 2023). SCLC, with its small cellular morphology, is distinguished by its rapid proliferative kinetics and propensity for swift metastasis to organs, such as the liver and brain, through lymphatic and hematogenous routes. Consequently, the prognosis of SCLC is poor, with a 5-year survival rate less than 7% (Siegel et al., 2022). In contrast, the two predominant histological subtypes of NSCLC are adenocarcinoma (ADC; approximately 50%) and squamous cell carcinoma (SCC; approximately 40%) (Davidson et al., 2013).

The high incidence and mortality rates of LC pose significant global health challenges. Therefore, understanding these factors is essential to improve patient outcomes. The incidence of LC largely mirrors smoking rates with a latency period of several decades. Consequently, smoking cessation is considered the most effective strategy to reduce the risk of LC. Other important risk factors include environmental exposure to radon, occupational carcinogens, and preexisting nonmalignant lung diseases. Addressing this challenge requires a comprehensive approach to the various treatment modalities available for managing the complexities of LC.

Therapeutic strategies for LC can be classified into five main categories: surgery, chemotherapy, radiotherapy, immunotherapy, and targeted therapy. Multimodal treatment approaches have been used to improve patient survival. However, owing to the challenges of early diagnosis, the overall 5-year survival (OS) rate of LC remains low at only 19%, and therapeutic outcomes are unsatisfactory (Bray et al., 2024). In recent years, targeted therapy for LC has undergone rapid evolution with significant advancements. Given the high mutation rates in LC, progress in genetic and biomarker detection has made personalized, targeted therapies for individual patients increasingly feasible (Zappa and Mousa, 2016). As the treatment landscape evolves, personalized approaches driven by molecular and genetic insights have become pivotal in improving patient outcomes. Advances in molecular biology hold promise for developing chemopreventive agents that prevent the onset of LC (Bilello et al., 2002). Notably, the phosphatidylinositol-3-kinase (PI3K)/protein kinase B (AKT)/mammalian target of rapamycin (mTOR) signaling (PAM) pathway has emerged as a key target for the development of effective therapies.

The PAM signaling pathway is a key driver of carcinogenesis and drug resistance in patients with solid tumors. Abnormalities in the PAM signaling pathway are present in approximately 50% of tumors, making it the most frequently activated signaling pathway in human cancers (Martini et al., 2014). This pathway is regulated by various upstream signaling proteins and plays a crucial role in modulating downstream effectors through interactions with compensatory signaling pathways, particularly the RAF/MEK/ERK pathway. The limited clinical success of targeted therapeutic agents highlights the importance of addressing alterations in the PAM signaling pathway when designing effective personalized treatment strategies (Ersahin et al., 2015). The use of inhibitors targeting key molecules within the PAM signaling pathway has garnered significant interest in targeted LC therapy. Indeed, several studies have developed promising inhibitors of this pathway, particularly for NSCLC (Fumarola et al., 2014). Several targeted therapies aimed at the PAM signaling pathway, including buparlisib (a PI3K inhibitor), MK2206 (an AKT inhibitor), sirolimus (an mTOR inhibitor), and perifosine (a dual PI3K/AKT inhibitor), are currently undergoing clinical trials for the treatment of LC (Moran et al., 2017; Primo N. et al., 2011; Novartis Pharmaceuticals, 2013a; AEterna Zentaris, 2006). Furthermore, several preclinical studies on natural compounds have garnered interest, with findings highlighting their potent inhibitory effects on the PAM signaling pathway (Zhang et al., 2021; Han et al., 2019; Niu et al., 2023). The high incidence and mortality rates of LC highlight the need for further research and treatment development.

In this review, we explored the role of the PAM signaling pathway in tumorigenesis and disease progression and reviewed recent advancements in preclinical studies and clinical trials targeting the PAM signaling pathway, focusing on the potential of natural products as therapeutic agents.

## 2 Biological characteristics of the PAM signaling pathway

**FIGURE 2:**
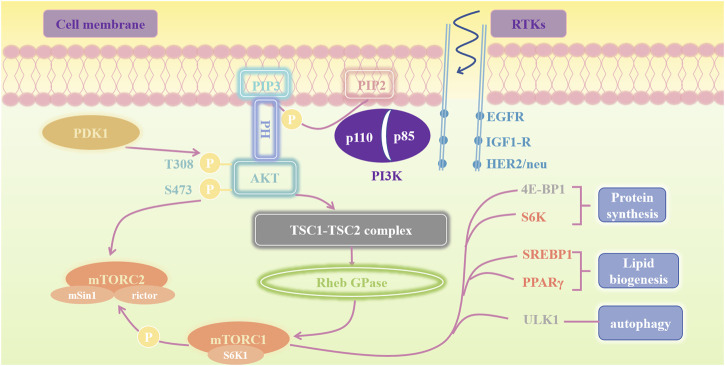
Schematic Representation of the PI3K/AKT/mTOR Pathway. This figure illustrates the PI3K/AKT/mTOR pathway involved in cell growth, protein synthesis, lipid biogenesis, and autophagy regulation. Upon activation of receptor tyrosine kinases (RTKs) such as EGFR, IGF1-R, and HER2/neu, PI3K is activated, converting PIP2 to PIP3. This leads to the activation of AKT via phosphorylation at T308 by PDK1 and S473 by mTORC2. Activated AKT regulates the TSC1-TSC2 complex, which controls Rheb GTPase, thereby activating mTORC1. mTORC1 promotes protein synthesis (via 4E-BP1 and S6K), lipid biogenesis (via SREBP1 and PPARγ), and autophagy regulation (via ULK1).

PI3K, an enzyme comprising a large family of lipid and serine/threonine kinases (Koundouros and Poulogiannis, 2020), is a heterodimeric protein comprising a p110 catalytic subunit and a p85 regulatory subunit (Figure 2) (Engelman et al., 2006). P110 catalytic subunits (p110α, p110β, p110δ, and p110γ) are encoded by PIK3CA, PIK3CB, PIK3CD, and PIK3CG, respectively (Fu et al., 2023). All p85 regulatory subunits (p85α, p85β, p55α, p55γ, p50α) are encoded by PIKR1 (Mazloumi Gavgani et al., 2018). Three types of PI3K (classes I, II, and III) have been identified in mammals. Class I PI3K is further divided into two subtypes: class 1A (p110α, p110β, p110δ) and 1B (p110γ), the most common type of cancer, is normally activated by RTKs, such as Epidermal Growth Factor Receptor (EGFR), insulin-like growth factor receptor (IGF1-R), and human epidermal growth factor receptor 2 (HER2/neu) (Higgins et al., 2016). Conversely, Class IB consists of one of the two regulatory subunits, p101 or p87, and the PIK3CG-encoded isoform, p110γ (Mazloumi Gavgani et al., 2018). Class II PI3K comprises three distinct enzymes: PI3K-C2α, PI3K-C2β, and PI3K-C2γ. In contrast, class III PI3K has only one known member, Vacuolar Protein Sorting 34 (Vps34, also known as PI3K-C3), the sole PI3 kinase expressed in all eukaryotes (Martini et al., 2014). PI3K signaling is one of the most commonly dysregulated pathways in cancer, promoting cell growth, proliferation, and survival (Koundouros and Poulogiannis, 2020). Activation of the receptor tyrosine kinase recruits PI3Kα to the plasma membrane, where it phosphorylates phosphatidylinositol 4,5-bisphosphate (PIP2) to phosphatidylinositol 3,4,5 triphosphate (PIP3). As a second messenger, PIP3 binds to and recruits various lipid-binding domains of downstream targets, such as the pleckstrin homology (PH), FYVE, phox (PX), C1, and C2 domains, to the cell membrane (Manning and Cantley, 2007). The PIK3CA mutation, which activates PI3K α, is one of the most common PI3K/AKT activation mechanisms in cancer. Distortion of NF1, MET, ERBB2, and RIT1 can also activate the PI3K signaling pathway (The Cancer Genome Atlas Research Network, 2014).

The AKT/mTOR signaling pathway is initiated by PI3K activation. AKT, also known as PKB, is a 57-kDa serine/threonine kinase that is a key mediator of growth factor-nduced cell survival. In the mammalian genome, three AKT isoforms have been identified: AKT1, AKT2, and AKT3. AKT1 is the primary subtype crucial in regulating cell apoptosis (Datta et al., 1999). AKT1 binds to PIP3 and facilitates the activation of 3-phosphoinositide-dependent protein kinase 1 (PDK1) and mTOR complex 2 (mTORC2). PI3K activation leads to the phosphorylation of two critical residues in AKT1: threonine 308 (T308) in the activation loop and serine 473 (S473) in the C-terminal hydrophobic motif (Alessi et al., 1996). The phosphorylation of these two residues is a critical step in the maximal activation of AKT1. The corresponding residues in AKT2 (T309 and S474) and AKT3 (T305 and S472) regulated this activation process. PDK1 plays a key role in AKT activation by phosphorylating AKT1 at T308 (Alessi et al., 1997; Stokoe et al., 1997). Maximal activation of AKT requires phosphorylation at S473 in the hydrophobic motif, and mTORC2 is the primary kinase responsible for this phosphorylation (Sarbassov et al., 2005). Although AKT retained some activity without S473 phosphorylation, its activity was significantly reduced. Phosphorylation of S473 stabilizes T308 phosphorylation and maintains AKT in its fully active state (Alessi et al., 1996; Yang et al., 2002). EGFR-Y1068 self phosphorylates to provide a docking site for SH2 containing adaptor proteins, directly activating the PI3K/AKT pathway to maintain cell survival (Rodrigues et al., 2000). Phosphorylation of EGFR at Y992, Y1148, and Y1173 sites leads to recruitment of SHC to activate the MAPK/ERK pathway, thereby promoting cell proliferation (Okabayashi et al., 1994). IGF1R promotes cancer cell proliferation and differentiation by inducing phosphorylation of PI3K and AKT (Hua et al., 2022).

mTOR is a serine/threonine protein kinase that is the primary catalytic subunit of two protein complexes, mTOR complex 1 (mTORC1) and mTORC2. mTOR was originally discovered as a cellular target of rapamycin and is involved in the checkpoint regulation of cell cycle regulation and regulates various biological processes, including cell proliferation, survival, autophagy, metabolism, and immunity (Saxton and Sabatini, 2017; Harwood et al., 2018). Inhibition of mTOR can effectively inhibit the growth, proliferation, cell cycle progression, migration, and invasion of NSCLC cells, while inducing apoptosis activation (Yang et al., 2020).

The mTORC1 complex, which comprises mTOR, mLST8, Raptor, and PRAS40, is highly sensitive to rapamycin, making it a key target for mTOR inhibitors, particularly first-generation inhibitors (Hay and Sonenberg, 2004).

mTORC1 and mTORC2 are intricately linked. mTORC1 exerts feedback inhibition on mTORC2 through S6K1, one of its substrates. Upon activation by mTORC1, S6K1 phosphorylates Rictor at T1135 and mSin1 at T86 and T398, leading to the disruption of mTORC2 integrity (Dibble et al., 2009; Julien et al., 2010; Liu et al., 2013). mTORC2 activates the IGF-IR–AKT axis, thereby upregulating mTORC1 (Yin et al., 2016). Phosphatase and tensin homolog (PTEN) is a negative regulator of mTOR, which inhibits signal transduction via the PI3K/AKT signaling pathway. Other regulatory factors include the tuberous sclerosis complex (TSC) comprising TSC1 (hamartin) and TSC2 (tuberin) (Tewari et al., 2022). AKT inactivates the TSC1-TSC2 complex, thereby promoting the activation of RHEB GTPase, activating mTORC1 (Inoki et al., 2002). Subsequently, active mTORC1 promotes protein synthesis by inactivating the translation inhibitor 4E-BP1 and activating the kinase S6K; (ii) induces lipid biogenesis by activating the transcription factors SREBP1 and PPAR; (iii) inhibits autophagy by blocking ULK1 (Laplante and Sabatini, 2012; Ma and Blenis, 2009). mTORC2 directly activates AKT via phosphorylation at Ser-473 site (Dong et al., 2014; Benetatos et al., 2017).

Previous studies have identified non-coding RNA (ncRNA) as an essential regulatory factor in the PAM signaling pathway (Yang et al., 2019). ncRNAs function as both upstream and downstream effectors in regulating the PI3K pathway and, directly or indirectly, targets multiple key components of this pathway, including PI3K, AKT, and mTOR. However, the precise mechanisms by which ncRNAs exert these effects remain to be fully elucidated (Fumarola et al., 2014; Liu et al., 2018; Hong et al., 2020).

## 3 Roles of the PAM signaling pathway

The PAM signaling pathway is a crucial intracellular signaling pathway that regulates various cellular processes, including cell growth, survival, proliferation, metabolism, apoptosis, invasion, and angiogenesis (King et al., 2015), and is one of the most commonly dysregulated pathways in cancers and pivotal in oncogenesis. Activation of the PAM signaling pathway can inhibit autophagy and apoptosis, which are typically associated with pro-survival effects (Luo et al., 2003; Xu et al., 2020). Most importantly, the excessive activation of key molecules within this pathway can unjustifiably promote cancer cell survival by inhibiting autophagy and apoptosis (Janku et al., 2018). The inhibition of this survival pathway induces autophagy and apoptosis in cancer cells (Yang et al., 2018).

The PAM signaling pathway is as a central signal transduction hub in response to extracellular stimuli, including insulin, insulin-like growth factor-1 (IGF-1), fibroblast growth factor (FGF), and epidermal growth factor (EGF) (Li and Wang, 2014). Chemokine receptor 9 (CCR9), inflammation-associated cytokine Toll-like receptor (TLR), and interleukin (IL)-6 are key upstream regulatory factors of the PAM signaling pathway (Harashima et al., 2012; Axanova et al., 2010; Wegiel et al., 2008). High expression levels of CCR9 are associated with increased tumor proliferation and metastasis (Chai et al., 2022). IL6 promotes tumor cell survival through PI3K/AKT and cyclin A1 (Wegiel et al., 2008). The upregulation of TLR-4 coexists with the downregulation of PI3K, AKT, and mTOR, associated with reduced cell damage (Kamel et al., 2020).

Genetic alterations targeting the PAM signaling pathway are closely associated with cancer development. For example, the loss of PTEN in somatic cells represent the second most common oncogenic mutations in the human genome, second only to malignant p53 mutations (Yin and Shen, 2008). In recent years, the rising incidence of malignant tumors has been linked to abnormal activation of the PAM signaling pathway (Aziz et al., 2018). Mutations or overexpression of PI3K can result in hyperactivation of this pathway, driving cancer cell proliferation and survival. IL-7 upregulates PI3K expression and promotes AKT and mTOR phosphorylation, activating the PAM signaling pathway (Jian et al., 2019). Abnormal expression of KIF2A promotes malignant transformation of cell carcinoma. Different analytical approaches focusing on antitumor miRNAs and their specific targeting of oncogenes are of great significance for identifying the molecular mechanisms underlying the pathogenesis of lung squamous cell carcinoma (Uchida et al., 2019). AKT is constitutively active in many cancers, promotes resistance to apoptosis, and enhances tumor survival (Shariati and Meric-Bernstam, 2019). mTOR is often hyperactivated in cancer, leading to increased protein synthesis, cell growth, and angiogenesis, all essential for tumor progression (Noser et al., 2023; Yang et al., 2023). Mutations in PI3K, loss of function of the PTEN tumor suppressor (a negative regulator of the pathway), and amplification or mutation of AKT can result in the constitutive activation of the PAM signaling pathway, driving uncontrolled cell proliferation and survival (Yehia et al., 2020). Owing to its pivotal role in cancer, the PAM signaling pathway is a major target for cancer therapies. Inhibitors targeting PI3K, AKT, and mTOR are undergoing clinical trials for various cancer types. However, drug resistance and feedback loops within the pathway remain major limitations that complicate the treatment strategies.

The PAM signaling pathway is involved in metabolic, cardiovascular, and neurodegenerative diseases (Ramasubbu and Devi Rajeswari, 2023; Tan et al., 2023). For example, activation of the PAM signaling pathway alleviates ischemia-reperfusion injury in diabetic cardiomyopathy (Tan et al., 2023). This pathway also regulates insulin signaling and glucose homeostasis, making it a key focus of diabetes research (Zheng et al., 2023). Furthermore, applying plant-derived secondary metabolites in managing and treating various neurological diseases through modulation of the PAM signaling pathway is a promising neuroprotective strategy (Fakhri et al., 2021).

Understanding the biological complexities of the PAM signaling pathway is essential for elucidating its role in cancer progression and resistance mechanisms. Building on this foundation, the following section discusses therapeutic implications, focusing on how these insights can be applied to develop targeted treatment strategies for LC.

## 4 PAM signaling pathway associated with targeted therapy

The PAM signaling pathway is one of the most frequently dysregulated networks in human cancers and plays a critical role in the proliferation and survival of LC cells. In LC cells, this pathway is often abnormally activated. For example, mutations in the PI3K gene or overexpression of AKT protein are common, which can cause uncontrolled proliferation, inhibited apoptosis, and enhanced migration and invasion ability of LC cells. Consequently, numerous drugs, most of which are small-molecule compounds, targeting these three key kinases---PI3K/AKT/mTOR have been developed and evaluated. Although these advances have contributed to significant progress, the effectiveness and future potential of these therapies require further exploration of specific inhibitors and their clinical applications.

In the following sections, we explore inhibitors targeting the PI3K, AKT, and mTOR components of the PAM signaling pathway, analyzing their mechanisms of action, outcomes of current clinical trials, and challenges in optimizing their use for LC therapy (Table 1) (Supplementary Images S1–S3). This in-depth analysis sheds light on how these small-molecule inhibitors shape the future of LC treatments.

**TABLE 1:** Ongoing clinical trials of several drugs targeting PI3K/AKT/mTOR signaling pathway in lung cancer.

Drugs	Target	Trail ID	Phase	Cancer stage	Comment
Buparlisib (BKM120)	Pan-class I	NCT01820325	phase II	NSCLC	PI3K pathway activation can be detected using ctDNA, it may not be the primary oncogenic driver in NSCLC
	PI3K	NCT01723800	phase I	NSCLC	None
		NCT02194049	phase I	SCLC	None
Pictilisib (GDC-0941)	pan-PI3K	NCT00974584	phase Ib	NSCLC	Combining pictilisib with various standard first-line treatment regimens is feasible for NSCLC patients
Eganelisib (IPI549)	PI3Kγ	NCT02637531	Phase 1/1b	NSCLC	Eganelisib doses of 30 and 40 mg once daily in combination with PD-1/PD-L1 inhibitors are safe
TQ-B3525	PI3Kδ/α	NCT05284994	—	NSCLC	None
MK-2206	AKT1, AKT2, AKT3	NCT01147211	phase I	NSCLC	None
Ipatasertib (GDC-0068)	pan-AKT	NCT04467801	phase II	NSCLC	None
Capivasertib (AZD5363)	AKT1, AKT2, AKT3	NCT02117167	phase II	NSCLC	Cells with dual AKT activation and RAS mutation may still inhibit 4E-BP1 even if AKT is inhibited
Everolimus (RAD001 or Afinitor)	mTOR	NCT00124280	phase II	NSCLC	RAD001, when combined with erlotinib and chemotherapy, may be an effective treatment for advanced NSCLC
Temsirolimus (CCI-779)	mTOR	NCT01396408	phase I	NSCLC	Good tolerability of tacrolimus at a dose of 15 mg per week when combined with chest radiation therapy
Itraconazole	VEGF, FGF, mTOR	NCT03664115	—	NSCLC	Itraconazole use was beneficial in NSCLC in terms of 1-year PFS and ORR which was not reflected by improvement in 1-year OS.
Aspirin	AKT, mTOR	NCT03543683	—	NSCLC	Aspirin exposure significantly promoted the apoptosis suggesting that aspirin may overcome Osimertinib resistance by promoting the apoptosis
Gedatolisib (PF-05212384)	PI3Kα, PI3Kγ, mTOR	NCT02069158	phase Ib	NSCLC	None
		NCT03065062	phase I	SCLC	None
BKM120 and RAD001	pan-class I PI3K and mTOR	NCT01470209	—	Lung cancer	None
MK2206 and erlotinib hydrochloride	AKT1, AKT2, AKT3 and EGFR	NCT01294306	phase II	NSCLC	The combination of MK2206 and erlotinib meets predetermined clinical activity criteria in EGFR wild-type NSCLC patients
Gefitinib and AZD5363	AKT1, AKT2, AKT3 and EGFR	NCT01570296	Phase Ib	NSCLC	None
		NCT00452244	phase II	NSCLC	The combination of gefitinib and simvastatin results in a higher RR and longer PFS

### 4.1 Inhibition of PI3K

Within the PAM signaling pathway, PI3K is a primary drug target for cancer treatment since its hyperactivity is strongly associated with tumor progression, enhanced tumor microvascular formation, and increased cancer cell invasiveness (Liu et al., 2020a). Over the past few decades, several pharmaceutical companies have developed PI3K inhibitors to target this key pathway (Sirico et al., 2023; Bertucci et al., 2023). Although PI3K inhibitors have demonstrated significant therapeutic efficacy against human cancers, acquired and intrinsic resistance limit their clinical efficacy (Raith et al., 2023).

#### 4.1.1 Buparlisib

Buparlisib (BKM120), a 2,6-dimorpholino pyrimidine derivative, is a novel, potent, highly selective inhibitor of class I PI3K with confirmed anticancer effects in various solid tumor models (Hwang et al., 2022). However, it has not been approved by the U.S. Food and Drug Administration (FDA) for the treatment of specific cancer types (Emory University, 2011). Experimental studies have shown little correlation between mutations in K-Ras, p53, LKB1, PTEN, EGFR, or CDKN2A and the sensitivity of cells to BKM120. Therefore, BKM120 effectively inhibits the growth of NSCLC cells, regardless of genetic mutations (Ren et al., 2012). BKM120 has a rapid and effective inhibitory effect on the PAM signaling pathway in human NSCLC cells (Ren et al., 2012).

In SCLC and NSCLC, BKM120 may inhibit tumor cell growth by blocking key enzymes required for cell proliferation. Administering PI3K inhibitors, such as BKM120, in combination with chemotherapeutic agents, such as carboplatin, pemetrexed disodium, cisplatin, and etoposide, may enhance tumor cell killing (NCT01723800, NCT02194049) (Medifind, 2012; University of California, Davis, 2014). The open-label, two-stage, phase II study BASALT-1 (NCT01820325) evaluated the efficacy of the pan-PI3K inhibitor BKM120 in patients with recurrent NSCLC and PI3K pathway activation (Novartis Pharmaceuticals, 2013b). These results indicate that although PI3K pathway activation can be detected using circulating tumor DNA (ctDNA), it may not be the primary oncogenic driver in NSCLC. Therefore, combination therapies involving PI3K inhibitors and other agents may be more effective than monotherapy (Vansteenkiste et al., 2015).

#### 4.1.2 Pictilisib

Pictilisib (GDC-0941) is an efficient PI3K selective inhibitor. When combined with trastuzumab (an antitumor drug), pictilisib demonstrated significant therapeutic effects against cancer cells *in vitro* and on tumors *in vivo* (Junttila et al., 2009). One study investigated the treatment of patients with advanced NSCLC using pictilisib in combination with paclitaxel and carboplatin (with or without bevacizumab) or pemetrexed and cisplatin (with or without bevacizumab). The authors demonstrated that combining pictilisib with various standard first-line treatment regimens is feasible for NSCLC patients, and preliminary findings have indicated encouraging antitumor activity (NCT00974584) (Soria et al., 2017; Stephen et al., 2009).

#### 4.1.3 Eganelisib

Eganelisib (IPI549) is a first-in-class, orally administered, highly selective PI3Kγ inhibitor that has demonstrated anti-tumor activity in preclinical studies, both as a monotherapy and in combination with programmed cell death protein 1/ligand 1 (PD-1/PD-L1) inhibitors. Eganelisib reshapes the tumor immune microenvironment and promotes cytotoxic T cell-mediated tumor regression without directly targeting the cancer cells (De Henau et al., 2016). The IPI-549–01 study was the first-in-human, multicenter, open-label, phase 1/1b dose-escalation trial.

The results indicated that eganelisib doses of 30 and 40 mg once daily in combination with PD-1/PD-L1 inhibitors were selected for further evaluation in Phase II trials (NCT02637531) (Hong et al., 2023; Infinity Pharmaceuticals, Inc., 2015).

#### 4.1.4 TQ-B3525

TQ-B3525, a novel PI3Kδ/α dual inhibitor developed by Zhengda Tianqing, induces cell apoptosis and inhibits the proliferation of malignant tumor cells by inhibiting the expression of PI3K protein and reducing the phosphorylation level of AKT protein (Li et al., 2024). TQ-B3525 selectively inhibits the PI3Kδ and PI3Kα subunits, overcoming drug resistance attributed to the upregulation of PI3Kα activity when PI3Kδ alone is inhibited. An ongoing single-arm, open-label, multi-cohort, multicenter clinical study is currently investigating the safety and efficacy of TQ-B3525 tablets combined with osimertinib for the treatment of patients with advanced NSCLC (NCT05284994) (Single-arm, 2022).

#### 4.1.5 Alpelisib

Alpelisib (BYL-719) is a potent, selective, orally active PI3Kα inhibitor with high efficacy in targeting PIK3CA-mutated cancersits anticancer activity against squamous cell LC cells with PIK3CA mutations *in vitro* and *in vivo* studies (Bonelli et al., 2015). BYL719 enhances the anticancer effects of gefitinib by inhibiting the p-AKT signaling pathway activated by PI3K/AKT in gefitinib-resistant NSCLC cells. The combination of BYL719 and gefitinib produced a synergistic effect on EGFR-mutant NSCLC cells via PI3K/AKT activation. Acquisition of PIK3CA mutations may contribute to cell proliferation and gefitinib resistance in NSCLC cells harboring EGFR mutations. Thus, combination therapy with gefitinib and BYL719 is a promising strategy to overcome EGFR-TKI resistance driven by PI3K/AKT activation (Yu Y. et al., 2023).

Candidate biomarkers for PI3K inhibitors have shown predictive value in preclinical models and exhibit tissue-specific changes in primary tumors (Spoerke et al., 2012).

### 4.2 Inhibition of AKT

Several drugs can specifically inhibit AKT protein, preventing excessive activation of downstream targets in the PAM signaling pathway. Ongoing clinical trials are investigating AKT inhibitors, such as capivasertib and ipatasertib, as monotherapy and in combination with other agents for treating advanced LC. In addition, MK-2206 has been evaluated in advanced solid and hematological malignancies. However, none of these drugs have received FDA approval. Notably, capivasertib is a promising new treatment option for these patients and is expected to gain Food and FDA approval in the near future (Alves and Ditzel, 2023).

#### 4.2.1 MK-2206

MK-2206 is a potent, orally active allosteric inhibitor of human AKT1, AKT2, and AKT3, with demonstrated preclinical antitumor activity. A phase I study investigated the combination of MK-2206 and gefitinib in NSCLC patients who failed prior chemotherapy and epidermal growth factor receptor tyrosine kinase inhibitors (EGFR-TKIs) (NCT01147211) (Lin et al., 2010).

#### 4.2.2 Ipatasertib

Ipatasertib (GDC-0068) is an orally effective, highly selective, ATP-competitive pan-AKT inhibitor that activates FoxO3a and NF-κB through AKT inhibition, leading to p53-independent activation of PUMA. Ipatasertib also induces apoptosis in cancer cells and inhibits tumor growth in xenograft mouse models (Sun et al., 2018; Blake et al., 2012).

Preclinical studies have suggested that ipatasertib enhances the therapeutic efficacy of chemotherapy and immunotherapy by modulating the PI3K–AKT signaling pathway. Consequently, a multicenter phase II study is currently underway to evaluate the combination of ipatasertib and docetaxel for the treatment of patients with metastatic or advanced NSCLC who have failed or are intolerant to first-line immunotherapy (NCT04467801) (Clinical Research and Trials, 2020).

#### 4.2.3 Capivasertib

Capivasertib (AZD5363), a novel pyrrolopyrimidine-derived compound, inhibits all AKT isoforms and is an effective ATP-competitive AKT inhibitor with an IC50 of <10 nM for all three AKT subtypes. Using a GI50 critical value of <3 μM, 23% of a large panel of cell lines are sensitive to capivasertib inhibition, with three-quarters of these cell lines harboring PIK3CA mutations, PTEN deletions, or HER2 amplification. KRAS is a negative predictive biomarker of the capivasertib response (Davies et al., 2012). SAFIR02_Lung is an open-label, multicenter, randomized, phase II trial. Using high-throughput genomic analysis as a tool for treatment decision-making, this trial aims to identify actionable genetic abnormalities in NSCLC (NCT02117167) (UNICANCER, 2014). Cells with dual AKT activation and *RAS* mutations may inhibit 4E-BP1 even if AKT is inhibited (Middleton et al., 2015).

### 4.3 Inhibition of mTOR

mTOR inhibitors were the first PAM-targeted drugs to enter clinical practice (Hudes et al., 2007). mTORC1 activation promotes the synthesis of proteins, lipids, and nucleotides while inhibiting autophagy, thereby enhancing cell survival, proliferation, and growth. In parallel, mTORC2 activation regulates protein kinases, including AKT, further supporting cell survival and proliferation (Sarbassov et al., 2005; Mao et al., 2022). Therefore, the functions of both mTORC1 and mTORC2 offer critical insights into the targeting of mTOR complexes to tumors. However, the effectiveness of some mTOR inhibitors may be limited by compensatory feedback loops that result in AKT activation (Machl et al., 2016).

#### 4.3.1 Sirolimus

Sirolimus is an effective and specific inhibitor of mTOR. In tissue culture studies, sirolimus at concentrations as low as 1 ng/mL inhibits mTOR signaling in cells. The first report on combining an mTOR inhibitor with radiotherapy in humans demonstrated that patients tolerated the combination well. Both clinical and animal data indicated that sirolimus can be safely combined with radiotherapy and cisplatin to treat LC (Sarkaria et al., 2007).

#### 4.3.2 Everolimus

Everolimus (RAD001 or Afinitor) is a rapamycin analog that functions similarly by acting as a conformational inhibitor of mTOR. RAD001 is an FDA-approved drug used to treat kidney cancer. This prevents cancer cell proliferation and renders them susceptible to cell death. RAD001 improves progression-free survival in patients with advanced renal cell carcinoma who previously received VEGF-targeted therapies (Motzer et al., 2008). Similar to rapamycin, everolimus induces AKT activation while inhibiting mTOR signaling in human cancer cells, including NSCLC cells, as well as in tumor biopsies (Wang et al., 2008; O'Reilly et al., 2006). This Phase II, nonrandomized, open-label, multicenter study evaluated the efficacy of everolimus monotherapy in patients with advanced NSCLC previously undergone no more than two chemotherapy regimens (NCT00124280) (Open Label, 2005). Research has demonstrated that when administered at a daily dose of 10 mg, everolimus possesses favorable tolerability and safety within its pharmacological class. Additionally, preclinical evidence suggests that combining EGFRi and mTOR inhibitors can have cumulative or synergistic effects in NSCLC by sensitizing cells to DNA-damaging agents via mTOR inhibition. This indicates that everolimus, when combined with erlotinib and chemotherapy, may be an effective treatment for advanced NSCLC (Soria et al., 2009).

#### 4.3.3 Temsirolimus

Temsirolimus (CCI-779) is an mTOR kinase inhibitor with antiproliferative and antiangiogenic properties. Temsirolimus activates autophagy and prevents cardiac function deterioration in animal models (Choi et al., 2012) and is a potential candidate for combination therapy with radiotherapy in NSCLC owing its established anti-proliferative and anti-angiogenic activities in multiple epithelial tumors and its non-overlapping mechanisms with radiotherapy (Waqar et al., 2014). A phase I study evaluating the combination of tacrolimus and chest radiotherapy in patients with NSCLC demonstrated good tolerability of tacrolimus at a dose of 15 mg/week when combined with chest radiotherapy (NCT01396408) (Orphanet, 2011).

### 4.4 Multiple targets inhibition

#### 4.4.1 Itraconazole

Itraconazole is an oral antifungal drug that has demonstrated anticancer effects in NSCLC by inhibiting angiogenesis. Circulating levels of angiogenic factors are associated with invasive tumor growth, metastasis, and prognosis in various solid tumors, including NSCLC. The FDA has approved itraconazole as an anti-angiogenic agent that targets factors such as vascular endothelial growth factor (VEGF) and FGF. Itraconazole directly disrupts the production of mitochondrial adenosine triphosphate (ATP), activating the adenosine monophosphate-activated protein kinase (AMPK) pathway and subsequent inhibition of the mTOR pathway. The results of the first randomized controlled trial evaluating itraconazole in combination with chemotherapy for newly diagnosed metastatic NSCLC demonstrated a significant improvement in overall response rate (ORR) compared with the control group. Additionally, the 1-year PFS in the itraconazole group was significantly improved, although this was not reflected in the 1-year OS (NCT03664115) (Mohamed et al., 2021; Asmaa et al., 2018).

#### 4.4.2 Aspirin

Aspirin, a nonsteroidal anti-inflammatory drug (NSAID), not only possesses classic anti-inflammatory properties but also exhibits chemopreventive effects against various human cancers, including colon cancer, LC, breast cancer, and leukemia, making it a promising anticancer agent (Ulrich et al., 2006). Autophagy occurs frequently during tumorigenesis and cancer chemotherapy, and strategies to stimulate or inhibit autophagy have been proposed as potential cancer therapies (Yue et al., 2014). In LC cells, combination therapy with aspirin and ABT-737 (a Bcl-2 inhibitor) induces a stronger autophagic response than either drug alone. Autophagy triggered by this combination shifts the role of p38 from cell protection to death promotion, with p38 acting as a switch between two distinct types of cell death: autophagy and apoptosis (Zhang et al., 2015). In an *in vivo* study using a lung tumor model induced by 4-(methylnitrosamino)-1-(3-pyridyl)-1-butanone (NNK) + lipopolysaccharide (LPS), nitric oxide-releasing aspirin (NO-aspirin) significantly reduced the number and size of lung tumors, as well as the expression of phosphorylated EGFR and AKT, and the pro-inflammatory molecules TNF-α and interferon-γ (Song et al., 2018).

Aspirin pretreatment disrupts the binding of the TATA-box and p300 at the initiation region of the mTOR promoter in cancer stem cells (CSCs), thereby inhibiting the binding of RNA polymerase II at these sites and suppressing mTOR gene transcription. Consequently, the downregulation of mTOR results in the phosphorylation of Akt at Ser473, leading to the activation of Gsk3β, which in turn causes the destabilization of Snail and β-catenin, thereby reversing the epithelial-to-mesenchymal transition (EMT) (Khan et al., 2019). GSK-3 β is a serine/threonine protein kinase that plays an important role in regulating the degradation of cyclin D1 by phosphorylating Thr-286 (Yang et al., 2015). The dephosphorylation of GSK-3 β leads to the phosphorylation of cyclin D1, which is then degraded (Cross et al., 1995). Osimertinib-resistant cells exhibited abnormal activation of the PI3K/AKT/BIM pathway, and the classic drug aspirin has been shown to effectively reduce AKT phosphorylation and BIM activation. Consequently, aspirin may attenuate the PI3K/AKT/BIM signaling pathway, promoting apoptosis in osimertinib-resistant cells (NCT03543683) (Chung-Shien et al., 2018).

#### 4.4.3 Gedatolisib

Gedatolisib (PF-05212384) is a potent dual inhibitor of PI3Kα, PI3Kγ, and mTOR, effectively targeting both mTORC1 and mTORC2 complexes. A Phase Ib, single-arm, open-label, dose-escalating study evaluated the safety, pharmacokinetics, and pharmacodynamics of gedatolisib in combination with carboplatin and paclitaxel in patients with NSCLC. The results indicated that this combination was tolerable, with preliminary efficacy observed, particularly in clear cell ovarian carcinoma (CCOC) (NCT02069158) (Colombo et al., 2021; Medi find, 2014). An open-label Phase I clinical trial is currently evaluating the safety of gedatolisib in combination with palbociclib in patients with advanced SCLC. Although the FDA has approved palbociclib for other indications, palbociclib for this specific disease or gedatolisib alone or combined with palbociclib as a treatment option awaits approval. Palbociclib is an oral drug that inhibits the cell cycle, preventing cell growth. Gedatolisib is thought to modulate cell growth and survival by controlling key signaling events within tumor cells, potentially slowing or halting tumor activity (NCT03065062) (Dana-Farber, 2017).

#### 4.4.4 Dactolisib

Dactolisib (NVP-BEZ235) is an orally active, dual pan-class I PI3K and mTOR kinase inhibitor that targets p110α, p110γ, p110δ, p110β, mTORC1, and mTORC2 (Maira et al., 2008). Dactolisib induces significant antiproliferative effects in transgenic mice with carcinogenic KRAS-induced NSCLC, as well as in NSCLC cell lines expressing carcinogenic KRAS. Although dactolisib therapy alone cannot induce apoptosis, dual PI3K/mTOR blockade effectively sensitizes NSCLC cells expressing oncogenic KRAS to the pro-apoptotic effects of ionizing radiation (IR) *in vitro* and *in vivo*. Therefore, combining dual PI3K/mTOR blockade with IR may benefit patients with NSCLC expressing oncogenic KRAS (Konstantinidou et al., 2009). An abnormal vascular structure in the LC leads to hypoxia, which limits the effectiveness of radiotherapy. Dactolisib improves tumor oxygenation and vascular structure, contributing to effective vascular normalization. The significant therapeutic benefit of combining dactolisib and irradiation highlights its potential importance in cancer treatment (Fokas et al., 2012).

### 4.5 Combined therapy

#### 4.5.1 BKM120 and RAD001

The combination of BKM120 and RAD001 has shown a synergistic inhibitory effect on LC cell growth *in vitro* and *in vivo* (NCT01470209) (Emory University, 2011; Wang et al., 2008; Sun et al., 2005). This combination not only eliminated RAD001-induced AKT and eIF4E phosphorylation but also enhanced the inhibitory effect on 4EBP1 phosphorylation and p21 induction (Ren et al., 2012).

Although this combination was tolerable when the doses of both drugs were reduced, it remained effective (Owonikoko et al., 2020).

#### 4.5.2 MK2206 and erlotinib hydrochloride

MK2206 and erlotinib hydrochloride (OSI-774) may inhibit tumor cell growth by blocking certain enzymes essential for cell proliferation. A phase II trial investigated the side effects and efficacy of MK2206 combined with erlotinib hydrochloride in treating patients with advanced NSCLC who previously responded to erlotinib hydrochloride but subsequently experienced disease progression (NCT01294306) (Primo N. et al., 2011). Studies have shown that the combination of MK2206 and erlotinib meets the predetermined clinical activity criteria in patients with wild-type EGFR NSCLC, necessitating further clinical investigation. Inhibition of the AKT pathway in wild-type EGFR NSCLC warrants additional clinical evaluation (Lara et al., 2015).

#### 4.5.3 Gefitinib and AZD5363

Gefitinib (ZD1839) is a potent, selective, and orally active EGFR tyrosine kinase inhibitor that inhibits EGF-stimulated tumor cell growth by blocking EGF-induced EGFR autophosphorylation in tumor cells. In addition to its inhibitory effects on cell proliferation, gefitinib induces autophagy and apoptosis, making it a valuable agent for cancer research, particularly for LC and breast cancer (Wakeling et al., 2002; Pedersen et al., 2005; Piechocki et al., 2008). A Phase Ib trial investigated the combination therapy of gefitinib and the oral class I PI3K inhibitor BKM120 in patients with advanced NSCLC, focusing on those with alterations in PI3K pathway molecules and known overexpression of EGFR (NCT01570296) (National Cancer Centre, Singapore, 2012). A randomized Phase II trial compared treatment with either gefitinib alone or combined with simvastatin to treat patients with advanced NSCLC (NCT00452244) (Ji-Youn et al., 2007). The authors demonstrated that, in patients with wild-type EGFR non-adenocarcinoma, the combination of gefitinib and simvastatin results in a higher response rate (RR) and longer progression-free survival (PFS) than gefitinib alone. Therefore, simvastatin may enhance the efficacy of gefitinib in this subgroup of gefitinib-resistant NSCLC patients (Han et al., 2011).

EGFR inhibitors are generally ineffective in most NSCLC cases with wild-type EGFR, necessitating new treatment strategies. AKT signal transduction plays a crucial role in mediating EGFR survival in NSCLC. Combining gefitinib and AZD5363 demonstrated synergistic growth inhibition in EGFR-mutant (HCC-827 and PC-9) and wild-type (NCI-H522, NCI-H1651) NSCLC cell lines. Therefore, dual inhibition of EGFR and AKT is a potential early treatment strategy for patients with both EGFR mutant and wild-type NSCLC (Puglisi et al., 2014).

#### 4.5.4 NVP-BEZ235 and AZD6244

AZD6244 is an effective, selective, orally administered MEK1/2 inhibitor. The combination of NVP-BEZ235 and AZD6244 enhanced both antitumor and anti-angiogenic effects. Studies have shown that combining selective MEK and PI3K/mTOR inhibitors can effectively inhibit the growth of gefitinib-resistant tumors caused by EGFR T790M mutation, MET amplification, and KRAS/PIK3CA mutations (Qu et al., 2014). This new treatment strategy may offer a practical approach to treating these patients.

Although several drugs targeting the PAM signaling pathway have been developed, research on natural products demonstrated promising potential.

The next section explores the application of natural products in targeting the PAM signaling pathway and examines their underlying mechanisms.

## 5 Natural products targeting the PAM signaling pathway

Recently, drugs isolated and purified from natural products have garnered considerable interest. As a component of traditional Chinese medicine (TCM) used in disease treatment, TCM offers several advantages, including minimal side effects, noninvasiveness, low cost, and broad accessibility to cancer therapy. Numerous studies have demonstrated the antitumor activity of various TCM ingredients, with clear benefits in reducing toxicity and enhancing efficacy when combined with other treatment modalities (Table 2) (Le-Xin et al., 2024; Yang et al., 2024).

**TABLE 2:** Preclinical investigation of several natural products targeting the PI3K/AKT/mTOR signaling pathway in lung cancer.

Natural products	Resources	Mechanism of actions
Napabucasin (NB)	Furanonaphthoquinone	NB induces apoptosis and autophagy in LC cells by directly targeting AKT and mTOR proteins
Ophiopogonin B (OP-B)	Radix Ophiopogon japonicus	OP-B inhibits phosphorylated AKT (p-AKT) at the Ser308 and Thr473 sites and significantly induces autophagy without triggering apoptosis
Fucoidan	Brown seaweed	Fucoid polysaccharides significantly inhibit the phosphorylation of PI3K and its downstream target AKT in a concentration- and time-dependent manner, while also inhibiting mTOR phosphorylation in a concentration-dependent manner
Sophflarine A (SFA)	Sophora flavescens	It inhibits NSCLC cell proliferation by inducing pyroptosis, and impairs migration, invasion, colony formation, and angiogenesis through PAM-mediated autophagy
Euphorbia hirta (Eh)	Euphorbiaceae	The application of Eh AgNPs significantly reduced the phosphorylation levels of p-PI3K, p-AKT, p-mTOR, and p70S6K
Emodin	Rheum palmatum and Polygonam multiflorum	Emodin downregulated PI3K/AKT pathway and thereby induced apoptosis

For example, quercetin, kaempferol, and isorhamnetin demonstrated anti-NSCLC effects by targeting AKT1 and EGFR. Among these compounds, isorhamnetin exhibits the strongest binding affinity to EGFR, with a binding energy of −6.79 kcal/mol. The primary interactions between isorhamnetin and EGFR involve carbon-hydrogen bonds and Pi-sigma, Pi-sulfur, and Pi-alkyl interactions. Isorhamnetin inhibits the migration and invasion of A549 cells, induces cell apoptosis and G1 phase arrest, and reduces expression of P-PI3K and P-AKT in A549 cells (Tang et al., 2024). A drug may play a crucial role through multiple signaling pathways due to its multiple targets. For instance, Lanatoside C induces G2/M cell cycle arrest and inhibits cancer cell growth by weakening the MAPK, Wnt, JAK-STAT, and PI3K/AKT/mTOR signaling pathways (Reddy et al., 2019).

### 5.1 Napabucasin

Napabucasin is a furanonaphthoquinone compound derived from plants of the Bignoniaceae family, with botanical sources including Tabebuia cassinoides (Rao and Kingston, 1982), Millettia versicolor (Fotsing et al., 2003), Ailanthus integrifolia (Kosuge et al., 1994), Ekmanian longiflora (Peraza-Sánchez et al., 2000), Newbouldia laevis (Eyong et al., 2015), and Handroanthus impetiginosus (Wagner et al., 1989). Previous studies have demonstrated that napabucasin and other compounds isolated from the roots of Ekmanianthe longiflora exhibit potent cytotoxicity against various cancer cell lines, including colon cancer, LC, and multidrug-resistant oral epidermoid carcinoma (Peraza-Sánchez et al., 2000). Napabucasin induces apoptosis and autophagy in LC cells by directly targeting AKT and Mtor. In the interaction with AKT, the key residues, Lys14, Glu17, Tyr18, and Arg23, in the PH domain play crucial roles in hydrogen bonding. In the NB-mTORC2 complex, napabucasin facilitated hydrophobic interactions with residues Leu2185, Lys2187, Glu2190, Asp2195, Ile2237, Trp2239, Val2240, Asn2343, Met2345, Leu2354, and Phe2358 and formed hydrogen bonds with Ile2356 and Asp2357. These results suggest that napabucasin disrupts the interactions between ATP complexes and mTOR catalytic targets. Inhibition of the AKT and mTOR pathways results in a reduction of the anti-apoptotic proteins Bcl-2, Mcl-1, and c-Myc, inducing apoptosis and inhibiting proliferation. This leads to autophagy, as evidenced by converting LC3B I to LC3B II (Petsri et al., 2022).

### 5.2 Ophiopogonin B

Ophiopogonin B, a bioactive compound derived from Radix Ophiopogon japonicus, is traditionally used in Chinese medicine to treat pulmonary diseases. NCI-H157 and H460 cells, representing the main subtypes of NSCLC originating from squamous and large cell carcinoma, respectively, have been used to investigate the effects of ophiopogonin B. In these cells, ophiopogonin B inhibited phosphorylated AKT (p-AKT) at the Ser308 and Thr473 sites and significantly induced autophagy without triggering apoptosis. Notably, in NCI-H460 cells, ophiopogonin B suppressed the PAM signaling pathway than in NCI-H157 cells, suggesting it as a potential inhibitor of the PI3K/AKT pathway for the treatment of NSCLC (Chen et al., 2013).

### 5.3 Fucoidan

Fucoidan is a sulfated polysaccharide extracted from brown seaweed and is primarily composed of L-fucose and sulfate groups with an average molecular weight of approximately 20,000 Da. Fucoid polysaccharides exhibit a range of biological activities, including antibacterial effects (Zapopozhets et al., 1995), antioxidant (Wang et al., 2009), anti-inflammatory (Choi et al., 2010), anticoagulant (Dürig et al., 1997), and anti-tumor activities (Aisa et al., 2005). Fucoid polysaccharides significantly inhibited the phosphorylation of PI3K and its downstream target AKT in a concentration- and time-dependent manner and inhibited mTOR phosphorylation in a concentration-dependent manner. In addition, two direct downstream targets of mTOR, 4E-BP1, and p70S6K, which are markers of mTOR activity, were significantly downregulated. In A549 LC cells, fucoid polysaccharides inhibited MMP-2 activity by suppressing the PAM signaling pathway, reducing cancer cell migration and invasion (Lee et al., 2012).

### 5.4 Sophflarine A

Sophflarine A, a novel alkaloid derived from Sophora flavescens, exhibits significant antiproliferative activity against NSCLC cells both *in vitro* and *in vivo*. Sophflarine A is characterized by a unique 6/8/6/6 four-ring system formed by the intramolecular half-bridge of 5,6-seco sophocarpine and inhibits NSCLC cell proliferation by inducing pyroptosis and impairs cell migration, invasion, colony formation, and angiogenesis through PAM-mediated autophagy. Sophflarine A also promotes reactive oxygen species (ROS) production by inhibiting the PAM signaling pathway and reducing p62 expression, thereby inducing autophagy and facilitating the conversion of LC3B-I to LC3B-II (Luo et al., 2023).

### 5.5 Euphorbia hirta

*Euphorbia hirta* is an herbaceous plant in the Euphorbiaceae family commonly found worldwide. *E. hirta* is well-known for its efficacy against various fungal and bacterial infections (Ogbulie et al., 2007) and contains secondary metabolites, including terpenoids, flavonoids, phenols, and essential oils. Currently, nanobiotechnology and nanomedicine are gaining increasing attention due to advancements in delivering targeted therapies that specifically destroy malignant cells (Gowda et al., 2013). Silver ions can inactivate invading pathogens and cancer cells, making them one of the most extensively studied metals for their activity against microbial pathogens and malignant cells (Iravani, 2011; Ebrahiminezhad et al., 2016). Over the past 20 years, silver nanoparticles (AgNPs) have garnered significant attention for their potent antiviral, antibacterial, antifungal, antiangiogenic, and anticancer properties, making them valuable for various biomedical applications (Lansdown, 2006). Additionally, AgNPs selectively destroy malignant cells while protecting normal cells through the controlled release of Ag ions (Jeong et al., 2014). In addition, some studies have shown that silver AgNPs selectively destroy malignant cells while protecting normal cells by releasing silver ions (Priya et al., 2011). This demonstrates that the application of Eh-AgNPs significantly reduces the phosphorylation of p-PI3K, p-AKT, p-mTOR, and p70S6K. The administration of Eh AgNPs specifically decreased the expression of PI3Kγ, without affecting other PI3K subtypes such as PI3Kα, β, and δ. These findings suggest that Eh AgNPs induce cell apoptosis by downregulating PI3Kγ, disrupting the PAM/p70S6K signaling pathway (Ramachandran et al., 2022).

### 5.6 Emodin

Emodin (1,3,8-trihydroxy-6-methylanthraquinone) is a naturally occurring anthraquinone derivative isolated from the roots and bark of many plants, fungi, and lichens, including Rheum palmatum and Polygonam multiflorum (Shrimali et al., 2013). Emodin significantly downregulates the expression of AKT, p-AKT, I κ B- α, p-I κ B- α, p65, p-p65, mTOR, and p-mTOR in the AKT signaling pathway, thereby promoting apoptosis of HL-60 cells (Zheng et al., 2007). In cancer cells overexpressing Her2/neu, treatment with emodin inhibited MAPK and PI3K/AKT dependent pathways, thereby suppressing cell growth and inducing apoptosis. Research has shown for the first time that treatment with emodin leads to blocking the binding of Her2/neu to Hsp90, intracellular redistribution, and enhanced ubiquitination, thereby promoting the proteasomal degradation of Her2/neu. This may represent a new approach for targeted therapy of Her2/neu overexpressing cancer (Yan et al., 2011).

## 6 The transduction of PAM signaling in immunotherapy

The PAM signaling pathway plays a pivotal role in the maturation, differentiation, recruitment, and survival of immune cells. The regulation of the immune system via the PAM signaling pathway is finely tuned, enabling the precise mobilization or inhibition of specific immune cell subsets through tightly controlled signaling mechanisms.

**FIGURE 3:**
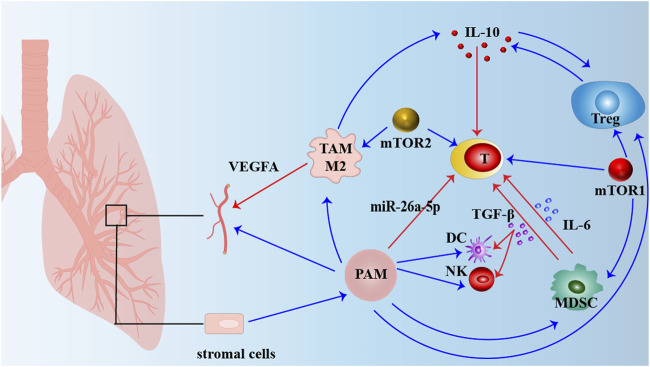
Interactions in the Tumor Microenvironment (TME) Affecting Lung Cancer Progression. This figure illustrates the interactions within the tumor microenvironment (TME) that contribute to lung cancer progression. Various components, including stromal cells, tumor-associated macrophages (TAM M2), pro-tumorigenic macrophages (PAM), regulatory T cells (Treg), dendritic cells (DC), natural killer (NK) cells, and myeloid-derived suppressor cells (MDSC), play significant roles in modulating immune responses and tumor growth. Stromal cells provide structural support and secrete growth factors that promote tumor proliferation. TAM M2 cells release VEGFA, stimulating angiogenesis and increasing blood supply to the tumor. Pro-tumorigenic macrophages (PAM) also interact with immune cells, promoting an inflammatory environment that favors tumor survival. Regulatory T cells (Treg) produce IL-10, which suppresses the activity of effector T cells and other immune cells, facilitating tumor immune evasion. The mTOR pathways (mTOR1 and mTOR2) regulate Treg function and T cell differentiation, contributing to an immunosuppressive environment that supports tumor growth. Myeloid-derived suppressor cells (MDSC) and dendritic cells (DC) secrete immunosuppressive cytokines such as IL-6 and TGF-β, which inhibit the anti-tumor activity of NK cells and T cells. miR-26a-5p further modulates these pathways, enhancing immune suppression. These complex interactions within the TME collectively create an environment that promotes immune escape, angiogenesis, and tumor progression.

The tumor immune microenvironment (TIM), a critical site for tumor growth, invasion, and immune evasion, comprises various immune cells, tumor-associated cells, and signaling molecules. The PAM signaling pathway plays a pivotal role in mediating the interactions between tumor cells and their surrounding microenvironment, particularly affecting immune cells and influences immune response, cell survival, and dysfunction (Figure 3).

### 6.1 Tumor-associated macrophage

The following outlines the interactions between key cells in the TIM and PAM signaling pathways: Tumor-associated macrophages (TAMs) are among the most abundant cellular components in the tumor microenvironment and typically exhibit an M2-type immunosuppressive phenotype (Guo et al., 2013). TAMs are a significant source of angiogenic factors that promote vascular growth, and subsequently accelerate tumor invasion and metastasis. TAMs contribute to angiogenesis by activating and releasing these factors, and TAM-derived VEGFA plays a crucial role in driving tumor-associated angiogenesis (Riabov et al., 2014). Therefore, TAM-mediated angiogenesis is a potential therapeutic target for malignant tumors. The PAM signaling pathway plays a key role in regulating the differentiation, survival, and function of TAMs. Excessive activation of the PAM signaling pathway drives the transformation of TAMs toward the M2 phenotype, enhancing their pro-tumor activities, such as the secretion of immunosuppressive cytokines like IL-10 and promoting angiogenesis, while simultaneously reducing the expression of M1-associated cytokines like TNF-α (Wang et al., 2018). Drugs that inhibit the PAM signaling pathway, such as PI3K inhibitors, may alter the phenotype of TAMs, transforming them from the M2 type to the antitumor M1 type, thereby enhancing the tumor immune response.

### 6.2 Tumor-infiltrating lymphocyte

Tumor-infiltrating lymphocytes (TILs) are T cells and other immune cells that infiltrate tumor tissues and are typically associated with better antitumor immune responses. This indicates the parameters of the immune response to tumors (Riabov et al., 2014). Numerous studies have demonstrated that the quantity and composition of TILs are associated with an improved response to ICI therapy in various solid tumors (Cristescu et al., 2018). Activation of the PAM signaling pathway in TILs can influence their antitumor capabilities. The regulation of the PAM signaling pathway can enhance the infiltration and antitumor activity of TILs, particularly CD8^+^ T cells (Zhuang et al., 2020). Compared with normal T lymphocytes, tumor-infiltrating T lymphocytes exhibited relatively low levels of miR-26a-5p. Experimental studies have shown that miR-26a-5p can inhibit the PAM signaling pathway, thereby reducing the ability of CD8^+^ tumor-infiltrating cells to eradicate tumors. Consequently, miR-26a-5p has emerged as a promising target for optimizing the efficacy of TIL therapy (Wang et al., 2023). For example, PI3Kδ-specific inhibitors can enhance the activity of TILs, thereby improving the immune-mediated clearance of tumors. Some tumor cells induce immunosuppressive signals by activating the PAM signaling pathway, which reduces the function (Chandrasekaran et al., 2021). This immune escape mechanism is a key focus of current research, with efforts to restore the antitumor activity of TILs by targeting the PAM signaling pathway.

### 6.3 Myeloid-derived suppressor cell

Myeloid-derived suppressor cells (MDSCs) are a critical class of immunosuppressive cells in the tumor microenvironment. They inhibit the function of effector T and NK cells, thereby promoting tumor growth (Budhwar et al., 2018). Granulocyte myeloid-derived suppressor cells (G-MDSCs) are the primary subpopulation of MDSCs and immature myeloid cells (IMCs) with immunosuppressive activity.

The role of suppressor of cytokine signaling 1 (SOCS1) is dependent on IFN-I signaling, which inhibits the activation of the PAM signaling pathway through a direct interaction with AKT. This suggests that the differentiation of immunosuppressive G-MDSCs involves a shift from immune activation to immune tolerance (Sun et al., 2022). The activation of the PAM signaling pathway enhances the immunosuppressive functions of MDSCs, leading to increased secretion of inhibitory cytokines, such as IL-6 and TGF-β, which suppress the anti-tumor activity of effector immune cells. Inhibition of the PAM signaling pathway can weaken the immunosuppressive function of MDSCs, thereby improving the effectiveness of tumor immunotherapy (Wu et al., 2020).

### 6.4 PI3K with immunotherapy

PI3K, particularly the PI3Kδ and PI3Kγ subtypes, predominant in white blood cells, are key regulators of immune homeostasis. Multiple studies have demonstrated that immunosuppressive regulatory T cells (Tregs) are highly dependent on PI3Kδ, as observed in human Tregs, mouse Tregs, and PI3Kδ-inactivated mouse models (Ahmad et al., 2017; Dong et al., 2019; Chellappa et al., 2019). PI3Kδ are involved in T cell receptor signaling, cell proliferation, and survival in Tregs. Notably, in a mouse model of LC, co-administration of PI3Kδ-specific inhibitors and tumor-specific vaccines reduced the number of inhibitory Tregs within the tumor microenvironment and increased the number of vaccine-induced CD8^+^ T cells, enhancing anti-tumor efficacy (Ahmad et al., 2017). Pharmacological inhibition of PI3Kδ effectively controls disease by significantly reducing the quantity, proliferation, and activation of CD25^+^ Tregs. However, this PI3Kδ-mediated reduction in Tregs did not improve CD8^+^ T cell function, as PI3Kδ inhibition also impairs T cell receptor signaling in CD8^+^ T cells, decreasing activation, effector differentiation, and proliferation (Hanna et al., 2019). Therefore, inhibiting PI3Kδ can suppress innate and adaptive immune systems, increasing patients’ susceptibility to severe infections. At the same time, the unique dependency of immunosuppressive regulatory T cells on PI3Kδ may explain the frequent occurrence of autoimmune conditions, such as pneumonia and colitis, in patients undergoing PI3Kδ inhibition (Tarantelli et al., 2021). Similar to PI3Kδ, the PI3Kγ subtype is active in lymphocytes. PI3Kγ, along with its regulatory subunits PIK3R5 and PIK3R6, is uniquely overexpressed in the bone marrow compartment, where PI3Kγ is the primary catalytic subunit for PI3K activity (Schmid et al., 2011). G protein-coupled receptors (GPCRs) activate p110γ through a Ras/p101-dependent mechanism, while receptor tyrosine kinases (RTKs) and Toll-like/IL-1 receptors (TLR/IL1Rs) activate p110γ via a Ras/p87-dependent mechanism. Once activated, p110γ promotes the inside-out activation of the integrin α4β1, facilitating the invasion of bone marrow cells into tumors. Pharmacological or genetic blockade of p110γ inhibits inflammation, tumor growth, and metastasis in both implanted and spontaneous tumor models (Schmid et al., 2011).

### 6.5 AKT with immunotherapy

AKT expression in most immune cells, both at the baseline and upon activation, underscores its critical role in immunity. AKT is essential for the regulation of both innate and adaptive immunity (Guerau-de-Arellano et al., 2022). AKT1 and AKT2, but not AKT3, promote terminal differentiation of CD8^+^ T cells while impairing the development of central and effector memory CD8^+^ T cell populations. These findings offer insights into adoptive cell transfer and vaccine-based cancer immunotherapies (Abu et al., 2015). In addition, AKT plays a critical role in CD4^+^ T cells by guiding helper T cell (Th) differentiation in a subtype-specific manner. AKT1 promotes antigen-specific Th1/Th17 responses by inhibiting the proliferation of thymes-derived T cells (Tregs). In contrast, AKT2 enhances tTreg proliferation *in vitro* and *in vivo*, while suppressing antigen-specific Th1/Th17 responses (Ouyang et al., 2019).

T cell activation through TCR and CD28 co-stimulation promotes the downstream induction of cytokines IL-2 and IFN-γ, mediated by T helper type 1 (Th1) cells in an AKT-dependent manner. In contrast, the regulation of IL-4 and IL-5 by T helper type 2 (Th2) cells occurs independently of AKT. FOXO is considered a tumor suppressor because they play an important role in inducing cell cycle arrest, DNA damage repair, and ROS clearance (Jiramongkol and Lam, 2020). AKT controls Treg homeostasis by inhibiting FOXO1 phosphorylation, enhancing Treg-mediated suppression (Ouyang et al., 2012). Treg cells expressing the transcription factor Foxp3 are essential for maintaining immune self-tolerance; however, excessive Treg activity can suppress antitumor immune responses. Low-dose expression of Foxo1 mutants has been shown to selectively deplete tumor-associated Treg cells, activate effector CD8^+^ T cells, and inhibit tumor growth without inducing autoimmunity. Inactivation of Foxo1 is crucial for migrating activated Tregs (aTregs), which play a key role in suppressing CD8^+^ T cell responses. FOXO4 activates the cell cycle dependent kinase inhibitor p27, which in turn inhibits the cell cycle dependent kinase (CDK) and blocks the G1 cell cycle progression in tumors. Silencing FOXO4 expression leads to an increase in cell cloning rate and migration enhancement (Yang et al., 2005). Modulating the FOXO signaling pathway in Treg cells offers a potential strategy for selectively disrupting tumor immune tolerance (Luo et al., 2016). Recent studies have demonstrated that Treg-mediated immune suppression is constrained in an AKT-dependent manner by PD-1 inhibition. Reduced signal transduction in the PI3K-AKT pathway is a key mechanism contributing to the enhanced suppressive capacity of PD-1-deficient Treg cells (Tan et al., 2021).

### 6.6 mTOR with immunotherapy

mTOR is the catalytic component of the mTORC1 and mTORC2 complexes, and its activation is essential for the proper activation and differentiation of effector CD4^+^ T cells (Zeng et al., 2013). These complexes collectively regulate cellular metabolism, the primary mechanism by which mTOR influences the immune system. mTORC1, in particular, plays a key role in regulating the differentiation of memory T cells (Araki et al., 2009); mTORC1 also partially sustains Treg cell function by inhibiting the mTORC2 pathway, linking immune signals from the TCR and IL-2 to adipogenesis pathways and functional adaptability. This highlights the central role of the Treg inhibitory activity in maintaining immune homeostasis and tolerance (Zeng et al., 2013). mTORC1 influences the effector response of CD8^+^ T cells, whereas mTORC2 regulates the memory of CD8^+^ T cells. mTORC2 inhibition leads to metabolic reprogramming driven by FOXO-mediated suppression of IL-15R expression, thereby enhancing the generation of CD8^+^ memory cells (Pollizzi et al., 2015). Importantly, mTORC1 and mTORC2 play direct roles in regulating innate immunity. mTOR is a key regulator of memory CD8^+^ T cell differentiation, and rapamycin, an immunosuppressive drug, has an immunostimulatory effect on the production of memory CD8^+^ T cells (Araki et al., 2009). mTORC1 regulates the production of inflammatory cytokines by inhibiting NF-κB, controlling monocyte/macrophage-mediated inflammation (Weichhart et al., 2008). mTORC2 complements this by modulating the chemotaxis of mast cells and neutrophils. Additionally, through FOXO1 inhibition, mTORC2 downregulates IL-12 production in dendritic cells and plays a key role in IL-4-dependent selective activation of M2 macrophages (Brown et al., 2011; Hallowell et al., 2017). mTOR regulates the expression of key inflammatory cytokines, including IL-10, IL-12, TGF-β, and TNF (Powell et al., 2012). mTOR-mediated inflammatory responses can also promote the recruitment of immune cells to TIM, which may exert antitumor effects or contribute to cancer cell growth, progression, and metastasis (Mafi et al., 2021). Activation of tumor-associated PAM signaling also promotes the expression of VEGF, a key mediator of angiogenesis and a chemokine that attracts immunosuppressive MDSCs and Tregs (Powell et al., 2012; Ostrand-Rosenberg and Fenselau, 2018; Goel and Mercurio, 2013). MDSCs accumulate in most cancer patients, promote tumor progression, suppress antitumor immunity, and impede the effectiveness of many cancer immunotherapies (Ostrand-Rosenberg and Fenselau, 2018).

In TIM, the PAM signaling pathway plays a crucial role in tumor immune escape and the efficacy of immunotherapy by regulating the activation, function, and inhibitory properties of various immune cells. Targeted therapies that inhibit the PAM signaling pathway not only directly suppress tumor cell growth but also enhance the effectiveness of immunotherapy by modulating the immune microenvironment. The advent of immunotherapy has transformed cancer treatments, marking the beginning of the immune era. Immune checkpoint inhibitors (ICIs) targeting PD-1/PD-L1 and cytotoxic T-lymphocyte antigen 4 (CTLA-4), along with cell-based therapies designed to target and destroy cancer cells, have demonstrated significant clinical efficacy.

Therefore, combining drugs that target the PAM signaling pathway with immunotherapies can significantly improve the survival rate of patients with LC. A randomized, prospective, multicenter, proof-of-concept Phase II clinical trial is currently evaluating the ORR of targeted therapy combined with the standard of care (SoC) in NSCLC (NCT04591431). Both immunotherapies, which leverage the body’s immune system to combat cancer, and targeted therapies are the cornerstones of personalized medicine. Ongoing research on gene mutations in tumor tissues or blood samples (e.g., circulating tumor cells [CTCs] or circulating free DNA [cfDNA]) is reshaping cancer treatment strategies. In the context of precision medicine, immuno-oncology is evolving into precision immuno-oncology with an emphasis on identifying predictive biomarkers that can optimize responses to ICIs (Annals of Oncology, 2020).

## 7 Challenges of the PI3K/AKT/mTOR inhibitors and future directions

PI3K inhibition is a key target for antitumor therapy. While several inhibitors, including copanlisib, alpelisib, idelalisib, duvelisib, and umbralisib, have been approved by the FDA, challenges remain regarding drug resistance, identification of sensitivity markers, and toxicity (Yu M. et al., 2023).

### 7.1 PI3K with side effects

Serious toxicities associated with PI3K inhibitors in clinical practice include hyperglycemia, skin reactions, diarrhea/colitis, pneumonia, and hypertension. Hyperglycemia typically occurs during the first two cycles of PI3K inhibitor treatment (Li et al., 2018; André et al., 2019). Hyperglycemia is regarded as an on-target effect of PI3K inhibitors and is linked to the critical role of the PI3K pathway in insulin signaling and glucose homeostasis (Juric et al., 2019). p110α and p110β regulate insulin-driven PI3K/AKT signaling pathway; inhibiting it can lead to hyperglycemia, rather than p110 δ and p110 γ (Moore et al., 2019). Additionally, the glucose-insulin feedback triggered by PI3K inhibitors is sufficient to reactivate PI3K signaling, thereby diminishing the effectiveness of the inhibitors (Horwitz et al., 2018). Skin reactions, such as rashes or papules, are among the most common toxicities observed with PI3K inhibitors in experimental studies. The PI3K/AKT signaling pathway is crucial in determining whether epidermal keratinocytes undergo differentiation or cell death (Liu et al., 2020b). Inhibition of this pathway suppressed keratinocyte proliferation and migration (Xenou and Papakonstanti, 2020). Additionally, activating the PAM signaling pathway inhibits autophagy and promotes inflammation in keratinocytes (Weidner et al., 2015). Diarrhea and colitis are common side effects of PI3K inhibitors, with severe cases often leading to treatment discontinuation (Curigliano and Shah, 2019). Histological examination via colonoscopy in patients with colitis revealed neutrophil infiltration, increased intraepithelial lymphocytes, and crypt cell apoptosis within the crypt epithelium (Wen et al., 2019).

The increased number of intraepithelial lymphocytes was predominantly CD8^+^ T cells, likely due to immune dysfunction. PI3Kδ inhibitors may disrupt B cell differentiation through immune dysregulation of Tregs, contributing to intestinal injury (Lynch et al., 2017). Fatal and severe pneumonia are common complications in patients receiving PI3K inhibitor treatments. Non-infectious pneumonia may be linked to the downstream inhibition of PI3K, whereas allergic and organizing pneumonia is more frequently observed in patients treated with mTOR inhibitors (Albiges et al., 2012). Pneumonia is also an immune-mediated disease, with a median increase in Th1-associated cytokines and chemokines observed in patient serum samples, including IFN‐γ and IL-6, -7, and -8 (Barr et al., 2016). Hypertension is one of the most common adverse events associated with acute vasoconstriction (Mateo et al., 2017). The PI3K/AKT signaling pathway plays a key role in regulating classical endothelial functions, including the modulation of vascular tone and recruitment of white blood cells to the vascular wall (Molinaro et al., 2019). p110γ is a key factor in regulating blood pressure. p110γ alleviates hypertension and reduces vascular inflammation by lowering peripheral resistance; conversely, it may contribute to developing hypertension and related target organ damage by modulating T cell function (Pridham et al., 2018).

Somatic mutations in PIK3CA also display unique patterns with respect to sex- and tissue-specificity (Benvenuti et al., 2008). Amplification of chromosome regions containing the PIK3CA gene has been identified in several human cancers, including ovarian, cervical, head and neck, and gastric cancers (Engelman et al., 2006; Shayesteh et al., 1999). PIK3CB undergoes a missense substitution (E633K) that enhances cell proliferation, transformation, and membrane targeting, reducing its dependence on Ras activation (Dbouk et al., 2013). Overexpression of p110γ can induce oncogenic transformation in cell cultures, and its increased expression promotes cell proliferation. However, downregulation via siRNA reduces proliferation, underscoring the critical role of p110γ in pancreatic cancer progression (Kang et al., 2006; Edling et al., 2010). p110δ is primarily implicated in blood cancers, and somatic mutations in its catalytic subunit (E1021K) are associated with recurrent infections and progressive airway damage (Angulo et al., 2013). High-throughput mutation analysis identified novel somatic mutations affecting p110γ (N66K, D161E, R178L, S348I, K364N, T503M, R542W, E602V, and E740K) and p110δ (V397A) across various tumor types, including breast cancer, LC, ovarian cancer, and prostate cancer (Kan et al., 2010).

The therapeutic efficacy of PAM signaling pathway inhibitors is limited and often accompanied by significant treatment-related toxicity, particularly when used in combination with standard therapies or other targeted drugs. Consequently, most PAM signaling pathway inhibitors are not suitable as mainstream treatments for tumors. Recent trends have shifted toward combining multiple drugs with other treatment modalities, such as surgery, hormone therapy, and additional antitumor agents. Future studies should identify reliable biomarkers for patient stratification based on cancer type and genetic characteristics, allowing for a more effective use of PI3K inhibitors. Since the full mechanism of action of PI3K inhibitors is yet to be fully elucidated, further research is required to better understand their advantages and limitations in the context of personalized cancer treatment.

### 7.2 AKT with side effects

The AKT pathway is crucial for regulating cell proliferation, survival, and metabolism, making AKT inhibition a potentially powerful antitumor strategy. However, dose-limiting toxicities and an incomplete understanding of the different AKT subtypes have hindered the successful pharmacological application of AKT inhibitors.

Treatment with AKT inhibitors may also result in adverse reactions, including gastrointestinal discomfort, skin reactions, metabolic disorders, abnormal liver function, hematological abnormalities, cardiac toxicity, and immunosuppression (Glaviano et al., 2023). AKT1 amplification has been detected in gastric cancer and is associated with cisplatin resistance (Liu et al., 2007). Somatic mutations in AKT1 have been identified in breast, colorectal, ovarian, LC, and bladder cancer (Knowles et al., 2009). AKT1 E17K is an activating mutation that causes constitutive localization of the protein to the plasma membrane, leading to hyperphosphorylation of serine-473 and threonine-308 in a growth factor-independent manner (Carpten et al., 2007). AKT2 amplification is frequently detected in various tumors, including ovarian, breast, colorectal, and pancreatic cancers, and is positively correlated with increased invasion and poor prognosis (Bellacosa et al., 1995; Parsons et al., 2005; Cheng et al., 1996). Selective activation of the AKT3 subtype combined with PTEN loss has been observed in 43%–60% of sporadic melanomas, indicating elevated levels of active AKT3 in the late stages of the disease (Stahl et al., 2004).

The pharmacological markers of AKT inhibitors have demonstrated incomplete targeted regulation. Currently, there are no approved biomarkers that can predict treatment response to specific AKT inhibitors before their use. Consequently, predicting which patients will experience adverse reactions due to AKT inhibition is challenging. However, the AKT E17K mutation is a promising biomarker, as clinical data have shown a correlation between this mutation and the response to the AKT inhibitor capivasertib (Hyman et al., 2017). Mutations that confer resistance to one AKT inhibitor may not necessarily confer resistance to others. For example, the clinically relevant AKT1 Q79K mutation can induce resistance to certain miRNAs but not MK-2206, highlighting the importance of understanding AKT genotypes when selecting appropriate treatments (Shi et al., 2014). Targeting AKT is a key focus of clinical oncology research, and future studies may improve its clinical efficacy by combining AKT inhibitors with synergistic cytotoxic drugs.

### 7.3 mTOR with side effects

Several mTOR inhibitors (mTORis) have been developed; however, only everolimus and temsirolimus have been approved for treating human tumors. Everolimus has shown acceptable safety in patients with neuroendocrine tumors, TSC-associated angiomyolipomas, renal cell carcinomas, and breast cancer. In patients with neuroendocrine tumors receiving everolimus monotherapy, grades 3–4 drug-related adverse events included stomatitis (9%), diarrhea (7%), infection (7%), anemia (4%), fatigue (3%), and hyperglycemia (3%). In renal cell carcinoma patients undergoing combination therapy with everolimus and lenvatinib, the most common grades 3–4 adverse events were constipation (37%) and diarrhea (20%) (Motzer et al., 2015; Janku et al., 2018). The most common grades 3–4 adverse reactions induced by temsirolimus included hypertriglyceridemia (44%), anemia (20%), hypophosphatemia (18%), lymphopenia (16%), hyperglycemia (16%), fatigue (11%), dyspnea (9%), neutropenia (5%), rash (5%), and pain (5%) (Kwitkowski et al., 2010). The clinical application of mTORi is often hindered by resistance driven by common molecular mechanisms across different drug categories. This suggests that co-targeting alternative pathways may be a more effective strategy than enhancing mTORi efficacy. Notably, preclinical and clinical data indicate that compared with other mTOR inhibitors, novel dual-space mTOR inhibitors can significantly promote cancer regression by inhibiting 4E-BP1 phosphorylation and reducing adaptive resistance by alleviating feedback inhibition of receptor tyrosine kinase (RTK) expression. Therefore, dual-space mTORC inhibitors are promising for treating cancers driven by activated mTORC1.

Additionally, developing new combination therapies may help to identify the molecular factors responsible for resistance to each drug class and improve patient selection for specific treatments. Optimizing the treatment sequences using different drugs may delay or overcome resistance to mTOR inhibitors.

### 7.4 miRNAs and lncRNAs

Long non-coding RNAs (lncRNAs), a class of ncRNAs longer than 200 nucleotides that regulate gene expression through various mechanisms, play critical roles in tumorigenesis (Hou et al., 2014). In recent years, many lncRNAs have been implicated in cancer progression by modulating the PAM signaling pathway. MALAT1, a well-known tumor-promoting lncRNA, competitively binds to microRNAs (miRNAs) such as miRNA-101 to relieve the inhibition of the PAM signaling pathway, thereby promoting tumor cell proliferation and migration (Goyal et al., 2021). Conversely, MEG3 is a tumor suppressor lncRNA that inhibits tumorigenesis by negatively regulating the PAM signaling pathway (He et al., 2021). The relative transcription level of colorectal cancer-related lncRNA colorectal cancer (OECC) was initially significantly upregulated in clinical LC tissues and cultured LC cells. Knocking down OECC in the LC cell line A549 led to a reduction in the mRNA levels of PI3K, phosphoinositide-dependent kinase-1, AKT, 5′-AMP-activated protein kinase, and endothelial nitric oxide synthase, while increasing the expression of tumor protein 53, neurofibromatosis protein 1, and Cullin-1 regulatory factors. These molecules are components of the PAM signaling pathway and/or involved in crosstalk (Zou et al., 2019). Notably, several genes located on chromosome 8q24 are associated with an increased risk of LC. For instance, CCAT1 promotes cell metastasis in lung adenocarcinoma via epithelial-mesenchymal transition and activates the Wnt signaling pathway in NSCLC (Lin et al., 2018; Xu et al., 2018; Hu et al., 2017). The novel lncRNA OECC, located on chromosome 8q24, regulates the proliferation and metastasis of human LC. lncRNA OECC is a novel regulatory factor in LC progression, offering novel insights into clinical treatment strategies. miRNAs are small, non-coding RNAs, typically 20–22 nucleotides in length, that regulate gene expression by binding to the 3′untranslated region of mRNAs. Many miRNAs have been shown to influence tumor cell behavior by regulating key proteins in the PAM signaling pathway. MiR-21, a well-known pro-tumor miRNA, directly inhibits PTEN expression, thereby activating the PI3K/AKT pathway and promoting tumor cell proliferation, migration, and survival (Wang et al., 2020). MiR-29 promotes tumorigenesis by enhancing mTOR signaling through inhibition of the TSC1/TSC2 complex (Hu et al., 2021). Conversely, miR-126 acts as a tumor suppressor by inhibiting the expression of the PIK3R2 subunit of PI3K, thereby suppressing the PI3K/AKT pathway and reducing cell proliferation (Hu et al., 2021). Research has demonstrated that the overexpression of microRNA-520a-3p significantly reduces the ratios of p-AKT/AKT, p-PI3K/PI3K, and Bcl-2/Bax, as well as the levels of mTOR, matrix metalloproteinase-2 (MMP-2), and matrix metalloproteinase-9 (MMP-9) in NSCLC. Conversely, inhibition of microRNA-520a-3p expression enhances cell proliferation, migration, and invasion, while suppressing apoptosis (Lv et al., 2018).

Overall, miRNAs and lncRNAs are promising therapeutic targets as regulatory molecules of the PAM signaling pathway. By designing miRNA mimetics or inhibitors or by interfering with lncRNA function, cancer-related signaling pathways can be modulated, offering new strategies for anti-cancer therapy. These approaches will facilitate the development of innovative cancer treatments.

Further research focusing on these molecules is expected to yield novel treatment options for cancer patients, and the outlook for LC treatment is optimistic. The PAM signaling pathway has demonstrated significant success in treating various tumors, and breakthroughs in LC therapy are anticipated. As more drugs targeting the PAM signaling pathway emerge, integrating multiple therapeutic approaches will play a crucial role in future treatment models. Multidisciplinary research may uncover increasingly effective strategies to gradually improve the survival rates of patients with LC. Additionally, advances in personalized precision medicine will offer more tailored and effective treatments, contributing to an overall improvement in the outcomes of this malignant disease.

## 8 Conclusion

In summary, the PAM pathway represents a critical target in the development of therapies for LC. Despite advances in identifying inhibitors and natural products targeting this pathway, challenges such as drug resistance, toxicity, and compensatory mechanisms persist. Addressing these issues requires a comprehensive understanding of the PAM pathway’s role in cancer progression and its interaction with other signaling networks.

Recent advancements in precision medicine have provided new opportunities for the personalized treatment of LC, with combination therapies showing promise in overcoming resistance. By integrating insights from molecular biology, clinical trials, and natural product research, future therapeutic strategies can be optimized to improve outcomes for patients with LC. Continued exploration of biomarkers, alongside the development of innovative inhibitors and combination regimens, holds the potential to advance treatment efficacy and mitigate existing limitations. The road ahead necessitates collaborative efforts in research and clinical practice to fully realize the potential of PAM pathway-targeted therapies.

## Glossary

LC: lung cancer
PI3K: phosphatidylinositol-3-kinase
AKT: protein kinase B
mTOR: mammalian target of rapamycin
IARC: International Agency for Research on Cancer
NSCLC: non-small-cell lung cancer
SCLC: small-cell lung cancer
ADC: adenocarcinoma
KRT7: keratin 7
SOX2: SRY-box 2
TTF1: thyroid transcription factor 1
OS: overall survival
PIP2: phosphatidylinositol 4,5-bisphosphate
PIP3: phosphatidylinositol 3,4,5 triphosphate
T308: threonine 308
mTORC1: mTOR complex 1
TSC: tuberous sclerosis complex
EGF: epidermal growth factor
IGF-1: insulin-like growth factor-1
FGF: fibroblast growth factor;
CCR9: Chemokine receptor 9
VEGF: vascular endothelial growth factor
AMPK: monophosphate-activated protein kinase
ATP: adenosine triphosphate
NSAID: nonsteroidal anti-inflammatory drug
ORR: overall response rate
LPS: lipopolysaccharide
CSCs: cancer stem cells
CCOC: clear cell ovarian carcinoma
IR: ionizing radiation
TCM: traditional Chinese medicine
SFA: Sophflarine A
ROS: reactive oxygen species
TIM: tumor immune microenvironment
TAMs: Tumor-associated macrophages
MDSCs: Myeloid-derived suppressor cells
G-MDSCs: Granulocyte myeloid-derived suppressor cells
RTKs: receptor tyrosine kinases
ICIs: Immune checkpoint inhibitors
CTCs: circulating tumor cells
MMP: matrix metalloproteinase
ADC: adenocarcinoma
SCC: squamous cell carcinoma
TTF1: thyroid transcription factor 1
PH: pleckstrin homology
PX: phox
IGF-1: insulin-like growth factor-1
FGF: fibroblast growth factor
EGF: epidermal growth factor
CCR9: Chemokine receptor 9
TLR: Toll-like receptor
IL: interleukin
FDA: Food and Drug Administration
AMPK: adenosine monophosphate-activated protein kinase
RR: response rate
Tregs: regulatory T cells
Th2: T helper type 2
RTKs: receptor tyrosine kinases
GPCRs: G protein-coupled receptors
Th1: helper type 1
CTLA-4: cytotoxic T-lymphocyte antigen 4
SoC: standard of care
mTORis: mTOR inhibitors
lncRNAs: Long non-coding RNAs
PAM: phosphatidylinositol-3-kinase (PI3K)/protein kinase B (AKT)/mammalian target of rapamycin (mTOR) signaling
