# Corrigendum: Targeting the PI3K/AKT/mTOR pathway in lung cancer: mechanisms and therapeutic targeting

**DOI:** 10.3389/fphar.2025.1591443

**Published:** 2025-04-24

**Authors:** Min Qiang, Zhe Chen, Hongyang Liu, Junxue Dong, Kejian Gong, Xinjun Zhang, Peng Huo, Jingjun Zhu, Yifeng Shao, Jinazun Ma, Bowei Zhang, Wei Liu, Mingbo Tang

**Affiliations:** ^1^ Department of Thoracic Surgery, The First Hospital of Jilin University, Changchun, China; ^2^ College of Clinical Medicine, Jilin University, Changchun, China; ^3^ Department of Molecular Biology, Max Planck Institute for Infection Biology, Berlin, Germany; ^4^ Laboratory of Infection Oncology, Institute of Clinical Molecular Biology, Christian-Albrechts-Universität zu Kiel and University Hospital Schleswig-Holstein, Kiel, Germany; ^5^ Department of Thoracic and Cardiovascular Surgery, Severance Hospital, Yonsei University College of Medicine, Seoul, Republic of Korea; ^6^ Department of General Surgery, Capital Institute of Pediatrics’ Children’s Hospital, Beijing, China

**Keywords:** PI3K/Akt/mTOR pathway, lung cancer, PI3K inhibitors, Akt inhibitors, mTOR inhibitors, natural products, combined therapy

In the published article, there was an error in the **Funding** statement. The funding statement in the article is written incorrectly as “Jilin Provincial Science and Technology Development Plan Project, 20220204115YY.” The correct statement is “Jilin Province science and technology development plan project:YDZJ202301ZYTS007”. The correct **Funding** statement appears below.

